# Prevalence and associated factors of suicidality in Japanese adolescents: results from a population-based questionnaire survey

**DOI:** 10.1186/s12887-020-02362-9

**Published:** 2020-10-06

**Authors:** Shinichiro Nagamitsu, Masakazu Mimaki, Kenshi Koyanagi, Natsuko Tokita, Yoriko Kobayashi, Ritsuko Hattori, Ryuta Ishii, Michiko Matsuoka, Yushiro Yamashita, Zentaro Yamagata, Takashi Igarashi, Paul E. Croarkin

**Affiliations:** 1 Department of Pediatrics and Child Health, Kurume University School of Medicine, 67 Asahi-machi Kurume, Fukuoka, 830-0011 Japan; 2 Department of Pediatrics, School of Medicine Teikyo University, Tokyo, Japan; 3 Nagasaki Prefectural Center of Medicine and Welfare for Children, Isahaya, Japan; 4 Department of Pediatrics, School of Medicine, Keio University, Tokyo, Japan; 5 Clinic of International University of Health and Welfare, Otawara, Japan; 6 Faculty of Health Science Naragakuen University, Nara, Japan; 7 Department of Neuropsychiatry, Kurume University School of Medicine, Kurume, Japan; 8 Department of Health Sciences, School of Medicine, University of Yamanashi, Kofu, Japan; 9 National Center for Child Health and Development, Tokyo, Japan; 10 Department of Psychiatry and Psychology, Mayo Clinic, Rochester, USA

**Keywords:** Suicide attempts, Adolescent, Child, Suicidal ideation, Cyberbullying

## Abstract

**Background:**

Suicide is the leading cause of death among Japanese adolescents. However, knowledge gaps regarding contemporary demographics and factors associated with suicidality among Japanese adolescents are a major concern. This study examined the prevalence of suicidality among Japanese adolescents and investigated associated factors.

**Methods:**

A population-based questionnaire survey investigating general health was administered to 22,419 adolescents aged 13–18 years. The 29-item questionnaire covered emotional status, family function, cyberbullying, suicidality, and stressors (e.g., relationships with parents/friends, school performance, and sexual identity). We conducted multiple logistic regression analysis to identify factors associated with suicidality in this population.

**Results:**

The prevalence of suicidal ideation was 21.6% in males and 28.5% in females, and that of attempted suicide was 3.5% in males and 6.6% and in females. Bullying and stress related to family relationships had the strongest associations with suicidality. Exposure to cyberbullying had the highest odds ratio for both junior high (3.1, 95% confidence interval [CI] 2.1–4.4) and high school students (3.6, 95% CI 2.5–5.3). Other factors significantly associated with suicidality were sex, emotional status, and stress about relationships with friends, sexual identity, school records, and academic course. Adolescents accessed a variety of resources to cope with stressors, with the Internet being the most common resource consulted.

**Conclusions:**

Suicidality is commonly experienced among Japanese adolescents. Although there are many associated risk factors, cyberbullying is of particular concern. Recognition of factors associated with adolescent suicidality will inform further research and suicide prevention efforts for healthcare providers and families.

## Background

The Promotion Council for Healthy Parents and Children 21 database (operated by Japan’s Ministry of Health, Labour and Welfare) recently identified a number of positive trends in adolescent behaviors and health [1]. For example, rates of smoking, alcohol consumption, sexually transmitted infections, induced abortions, and obesity have decreased in recent decades. However, the suicide rates among young people aged 10–14 and 15–19 years rose between 2002 (0.8 and 7.5 per 100,000, respectively) and 2012 (1.3 and 8.5 per 100,000, respectively) [2]. The prevalence of suicidal ideation and suicidal attempts has been reported in various countries, although the reported ranges differ. In some Western countries, the reported prevalence of suicidal ideation was 12.1–22.0% and that of suicidal attempts was 1.0–7.6% [3–7]. However, low- and middle-income countries showed higher prevalence rates [8–10], and some Asian countries had equal or lower prevalence rates than those reported for Western countries [11–13].

Prior research examined factors associated with suicidal ideation or attempts among adolescents [6, 7, 13–21]. The majority of youth with suicidality have preexisting psychiatric disorders. Saffer et al. [17] reported that lower parental bonding may be an important risk factor for youth suicidal behavior. Other studies suggested that adolescents who were either victims or perpetrators of bullying had an increased risk for suicide [14, 16]. Academic stressors and conflicted feelings about sexual identity are other putative risk factors for suicide among adolescents [18]. Recently, several cross-sectional studies showed associations between personal/social risk factors and suicidality among Japanese adolescents. *Hikikomori* (prolonged, severe social withdrawal and isolation), preference for solitude, low body mass index, appetite loss, violence, and psychotic-like experiences have been proposed as significant risk factors for Japanese adolescents [20–26]. Some meta-analyses have identified risk factors that were significantly associated with higher odds of suicidal ideation and attempts in adolescents, including stressful life events, bullying victimization, and peer victimization [14, 27–29]. Suicide is the leading cause of death among Japanese adolescents, and effective strategies are required to prevent suicide in this population. The Japanese government officially announced that poor school academic records and stress about academic courses were the most significant causes of suicide among school students [30]. However, multiple logistic regression analysis is necessary to identify individual and environmental factors associated with suicidality among adolescents.

The present study aimed to examine the prevalence of suicidal ideation and attempts among Japanese junior high-school and high school students. We also examined correlations between suicidal ideation or attempts and potential associated factors, including sex, age, grade, family structure, family function, emotional status, cyberbullying, number of friends, stressors (e.g., relationships with friends, relationships with parents, school records, academic course, sexual identity), and geographic region. Broadly, the findings may inform public health initiatives focused on adolescent health. We hypothesized that the prevalence of suicidality in our study would be similar to that found internationally, and bullying would be associated with suicide attempts.

## Methods

### Participants

Data for this study were drawn from a Promotion Council for Healthy Parents and Children survey conducted in November–December, 2016. Six education committees approved representative schools to participate in that survey. In total, we analyzed data for 22,419 students from 36 public junior high schools, five private junior high schools, 10 public high schools, and six private high schools. Participating junior high school students were aged 13–15 years (grades 7–9) and high school students were aged 16–18 years (grades 10–12). The participating schools were located in four urban cities (population over 1 million), four suburban cities (population around 300,000), and two rural areas (population around 50,000). These areas covered much of Japan, including Tokyo, Kyoto, Fukuoka, Tochigi, Aichi, and Nagasaki prefectures.

### Questionnaire content and survey procedure

The Promotion Council for Healthy Parents and Children 21 (Second Phase) reviewed and approved the questionnaire and survey process. The 29-item questionnaire covered: general information (age, sex, school grade, number of siblings, sleep habits, and Internet use); general feelings (emotional status and suicidality); family function; bullying (school or cyber); general stressors (e.g., relationships with family/friends, sexual identity, pregnancy, school bullying, and substance use) and stress about future events (e.g., marriage and raising children). We also examined resources that participants consulted to cope with stressors. They were asked to select the most useful single resource for each stressor from four options (parents, friends, school teacher/s, and the Internet). Table 1 presents examples of survey questions. Participants’ parents were informed about this questionnaire survey by letter, and the participating schools obtained passive informed consent from participants. The questionnaire was distributed directly to participating adolescents. Their class teachers explained the purpose of the study, and the anonymous self-administered questionnaire was completed during class time. Participants placed their completed questionnaire into a sealed envelope. Their teachers then collected the envelopes and sent them to Kurume University.

**Table 1:** Questionnaire items (sample)

Q1 What is your school grade?	
Q3 What is your sex?	
Q4 How many siblings do you have?	
Q8 How many friends do you have? (very many, many, not so many, very few, unknown)	
Q10 How often do you feel happy? (always, often, sometimes, rarely, never)	
Q11 How often do you feel well? (always, often, sometimes, rarely, never)	
Q12 How often do you feel lonely? (always, often, sometimes, rarely, never)	
Q13 How often do you talk with your family? (always, often, sometimes, rarely, never)	
Q14 Have you ever experienced cyberbullying? (yes, no)	
Q15 In the past year, have you seriously considered killing yourself? (no, sometimes, always, attempted suicide, no idea)	
Q17 Do you have stressors about the following: body image; secondary sexual characteristics; friends; conventional bullying; parents; siblings; school records; opposite sex; sexual identity or intercourse; sexually transmitted infections; induced abortion; contraception; marriage; pregnancy, child care; physical illness; academic course; alcohol, smoking, or substance use?	

### Statistical procedure and analysis

The survey data were used to determine the prevalence of suicidal ideation, suicide attempts, and potential associated factors among Japanese adolescents. To examine ideas about suicidality, participants were asked, “In the past year, have you seriously considered killing yourself?” (Q15). Participants were requested to select one of five response choices (never, sometimes, always, have attempted suicide, and no idea). We defined participants who selected “never” as having no suicidality, those that selected either “sometimes” or “always” as having suicidal ideation, and those who selected “have attempted suicide” as suicide attempts. To minimize the invasive effects of the survey on participants, responses for suicidal ideation and suicidal behaviors (suicide attempts) were listed in one answer column. We examined factors potentially associated with suicidality: grade (Q1); sex (Q3); number of siblings (Q4); number of friends (Q8); happiness (Q10); wellness (Q11); loneliness (Q12); amount of family conversation (Q13); experience of cyberbullying (Q14); and stressors (Q17). In Q17, participants were asked whether they had experienced specific stressors: relationships with friends; school bullying; relationships with parents; school records; relationships with the opposite sex; sexual identity; academic course; and tobacco or substance use. Each item in Q17 was listed as a noun in the questionnaire, and participants answered either “yes” or “no.” Geographic region was also examined as a factor potentially associated with suicidality.

We first determined the prevalence of suicidality by grade and sex. Then, we counted the numbers of stressors and calculated the ratio of consultation resources for each stressor. Univariate logistic regression analysis was used to examine factors potentially associated with suicidality among adolescents, with respect to both ideation and attempts. Separate analyses were conducted for junior and high school participants because we speculated that factors associated with suicidality could differ between junior high school and high school students. We conducted multiple logistic regression analysis to estimate odds ratios (OR). Potential confounders (e.g., grade, sex, number of siblings, number of friends, and stressors, as listed in Table 1) were included in the model as covariates.

## Results

### Participants

In total, there were 22,419 respondents (48% female) aged 13–18 years. There were 13,285 junior high and 9134 high school students. Notably, adolescents absent from school at the time of the survey were unable to participate. Participant numbers in each grade were: 4371 (7th grade); 4486 (8th grade); 4396 (9th grade); 3683 (10th grade); 3024 (11th grade); 2396 (12th grade); and 63 (grade undetermined). The distribution of participants’ locations and school types is presented in Table 2. There were no participants from private schools in rural areas.

**Table 2:** Participants’ geographic location and school type

	Junior high school (*n* = 13,285)		High school (*n* = 9134)		Total (*N* = 22,419)	
School type	Public	Private	Public	Private	Total	
Region						
Urban	746	1957	1304	3346	7353	
Suburban	8449	162	1923	803	11,337	
Rural	1971	162	1758	0	3729	

### Number of participants experiencing cyberbullying and stressors

There were 402 (1.8%) participants with experience of cyberbullying. The numbers of participants that reported each stressor type were: relationships with friends, 5381 (24.0%); school bullying, 573 (2.6%); relationships with parents, 2062 (9.2%); school records, 13,391 (59.7%); relationships with the opposite sex, 2383 (10.6%); sexual identity or intercourse, 553 (2.5%); academic course, 13,477 (60.1%); tobacco use, 363 (1.6%); and substance use, 167 (0.7%).

### Prevalence of suicide ideation and suicide attempt

The overall prevalence of suicidal ideation was 25.7%, and that of attempted suicide was 5.4%. Almost twice as many female adolescents had made a suicide attempt than males (6.6% vs. 3.5%). Notably, we observed little difference in the prevalence of suicide attempts in the 7th grade (Table 3).

**Table 3:** Prevalence of suicide ideation and suicide attempts

		n	None		Suicidal ideation		Suicide attempt	
Total		22,419	15,245	68.0%	5765	25.7%	1206	5.4%
Grade	7th	4371	3011	68.9%	1123	25.7%	203	4.6%
	8th	4486	3088	68.8%	1130	25.2%	233	5.2%
	9th	4396	3043	69.2%	1054	24.0%	261	5.9%
	10th	3683	2492	67.7%	961	26.1%	191	5.2%
	11th	3024	1990	65.8%	836	27.6%	176	5.8%
	12th	2396	1595	66.6%	648	27.0%	138	5.8%
Sex	Male	8907	6606	74.2%	1925	21.6%	309	3.5%
	Female	13,454	8613	64.0%	3830	28.5%	893	6.6%

The numbers and percentages of participants who selected “no idea” for suicidal question do not appear in the table. The numbers of participants with missing values for each factor likewise do not appear

### Consultation resources for stressors

**Fig. 1:**
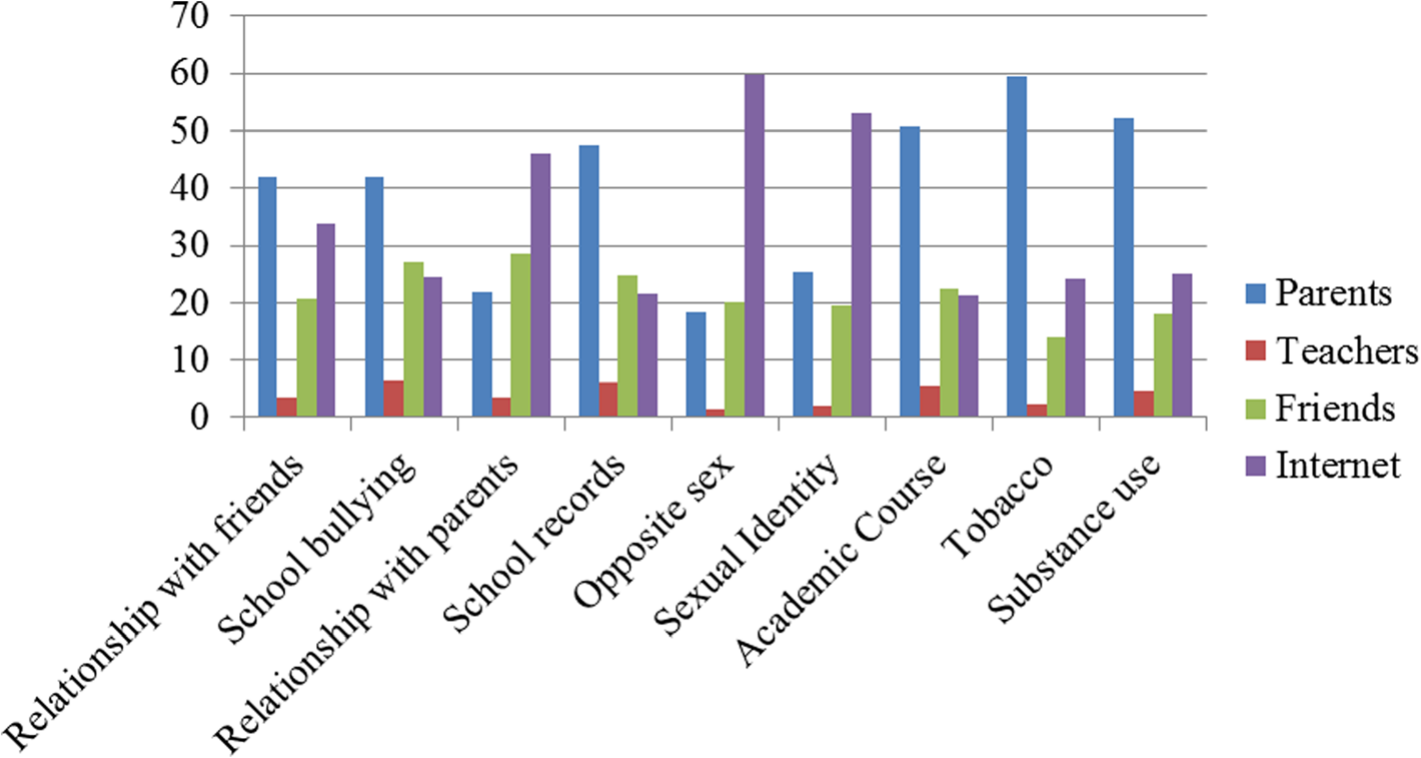
Percentages of adolescent respondents’ preferred resources by stressor type

Figure 1 shows the proportions of preferred resources participants’ selected for various stressors. They used different resources according to the type of stressor. Many participants indicated they consulted the Internet as a resource—even for matters concerning parent relationships. Notably, few adolescents recorded teachers as resource for addressing stressors.

### Multivariate regression analysis

Table 4 shows the results of univariate and multivariate logistic regression analysis. In the univariate logistic regression analysis, all covariates except school grade and number of siblings (junior high school students) were significantly associated with suicidality. The factors that showed high ORs were: experience of cyberbullying (junior high school students OR 6.5, 95% confidence interval [CI] 4.7–8.8; high school students OR 5.6 95% CI 4.0–7.7), stress about school bullying (junior high school students OR 5.3, 95% CI 4.3–6.4; high school students OR 8.9 95% CI 5.2–15.4), and stress about relationships with parents (junior high school students OR 5.0, 95% CI 4.4–5.6; high school students OR 4.2, 95% CI 3.6–4.9).

**Table 4:** Logistic regression analysis in junior high and high school children

			Junior high school children						High school children			
Variables	Crude OR	95%CI	*p* value	Adjusted OR^a^	95%CI	*p* value	Crude OR	95%CI	*p* value	Adjusted OR^a^	95%CI	*p* value
Grade	0.99	0.95–1.04	0.685	1.00	0.95–1.06	0.972	1.04	0.98–1.10	0.192	1.03	0.96–1.09	0.461
Sex	1.75	1.62–1.89	< 0.001	1.61	1.47–1.77	< 0.001	1.42	1.29–1.57	< 0.001	1.67	1.48–1.88	< 0.001
Number of siblings	0.95	0.85–1.05	0.311	1.06	0.93–1.20	0.412	0.76	0.70–0.89	< 0.001	0.86	0.75–0.99	< 0.05
Number of friends	1.40	1.36–1.44	< 0.001	1.05	1.01–1.09	< 0.05	1.31	1.26–1.36	< 0.001	1.05	1.00–1.10	< 0.05
Happiness	2.00	1.92–2.07	< 0.001	1.51	1.44–1.58	< 0.001	1.93	1.85–2.02	< 0.001	1.48	1.40–1.57	< 0.001
Wellness	1.74	1.68–1.80	< 0.001	1.24	1.18–1.29	< 0.001	1.72	1.64–1.79	< 0.001	1.28	1.22–1.35	< 0.001
Loneliness	0.51	0.50–0.53	< 0.001	0.69	0.66–0.72	< 0.001	0.51	0.49–0.53	< 0.001	0.65	0.62–0.68	< 0.001
Talk with family	1.41	1.36–1.46	< 0.001	1.15	1.10–1.20	< 0.001	1.37	1.31–1.43	< 0.001	1.11	1.05–1.17	< 0.001
Experience of cyberbullying	6.45	4.74–8.78	< 0.001	3.05	2.12–4.39	< 0.001	5.58	4.03–7.73	< 0.001	3.64	2.51–5.30	< 0.001
Stressors about												
Relationship with friends	3.68	3.39–4.00	< 0.001	1.57	1.42–1.74	< 0.001	3.05	2.75–3.38	< 0.001	1.37	1.21–1.55	< 0.001
School bullying	5.28	4.34–6.42	< 0.001	1.88	1.49–2.37	< 0.001	8.94	5.18–15.43	< 0.001	2.62	1.40–4.90	< 0.005
Relationship with parents	4.97	4.39–5.63	< 0.001	2.10	1.81–2.44	< 0.001	4.20	3.62–4.87	< 0.001	2.12	1.78–2.53	< 0.001
School records	1.79	1.65–1.93	< 0.001	1.24	1.13–1.37	< 0.001	1.56	1.43–1.71	< 0.001	1.20	1.07–1.34	< 0.005
Relationship with opposite sex	3.02	2.69–3.39	< 0.001	1.82	1.58–2.09	< 0.001	2.07	1.81–2.36	< 0.001	1.35	1.15–1.59	< 0.001
Sexual identity or intercourse	3.84	3.03–4.86	< 0.001	1.38	1.03–1.84	< 0.05	3.78	2.91–4.92	< 0.001	2.18	1.58–3.01	< 0.001
Academic course	1.72	1.59–1.86	< 0.001	1.18	1.07–1.30	< 0.001	1.51	1.38–1.66	< 0.001	1.14	1.02–1.28	< 0.05
Tobacco use	2.44	1.91–3.10	< 0.001	1.34	0.95–1.88	0.098	2.70	1.75–4.15	< 0.001	1.25	0.72–2.17	0.438
Substance use	2.20	1.56–3.11	< 0.001	0.77	0.47–1.27	0.304	4.37	1.98–9.67	< 0.001	0.79	0.28–2.21	0.650
Region	0.86	0.80–0.91	< 0.001	0.94	0.87–1.01	0.083	1.10	1.04–1.16	< 0.001	1.19	1.11–1.27	< 0.001

*OR* odd ratio, ^a^ Multiple logistic regression analysis including all variables listed in this table

In the multivariate logistic regression analysis adjusted for confounding variables, most covariates were significantly associated with suicidality in junior high school students, with the exception of grade, number of siblings, stress about tobacco/substance use, and geographic region. By contrast, all factors except grade and stress about tobacco/substance use were significant among high school students. The factors that showed high adjusted ORs among junior high school students were: experience of cyberbullying (OR 3.1, 95% CI 2.1–4.4); stress about relationships with parents (OR 2.1, 95% CI 1.8–2.4); and stress about school bullying (OR 1.9, 95% CI 1.5–2.4). Among high school students, the factors that showed high adjusted ORs were: experience of cyberbullying (OR 3.6, 95% CI 2.5–5.3); stress about bullying (OR 2.6, 95% CI 1.4–4.9); stress about sexual identity or intercourse (OR 2.2, 95% CI 1.6–3.0); and stress about relationships with parents (OR 2.1, 95% CI 1.8–2.5).

## Discussion

The present study examined the prevalence of suicidality among Japanese adolescents and associated factors. This study was characterized by nationally representative large-scale data that covered urban, suburban, and rural areas in Japan. We analyzed associated factors related to general information about participants, their feelings, family function, bullying, and various stressors.

The overall prevalence of suicidal ideation and suicide attempts among Japanese adolescents was 25.7 and 5.4%, respectively; these rates were similar to those observed in Western countries [3–7]. The prevalence was also consistent with previously reported figures for Japan [31–33]. School grade and geographic region did not appear to be associated with suicidality. However, a number of characteristics and perceptions appeared to be associated with suicidality in this sample. The strength of this cross-sectional survey was that we simultaneously analyzed a number of potential factors associated with adolescents’ suicidality, including environmental factors and related stressors. Prior experience of cyberbullying was identified as the most significant associated factor. We also identified school bullying, and stress about relationships with parents, relationships with the opposite sex, and sexual identity or intercourse as having high ORs.

Associations between bullying (cyber or school) victimization and suicidal behavior have been reported over the past decade, and the growing number of reports is of particular concern [34–38]. Psychological distress from cyberbullying is more severe than that associated with school traditional bullying, as it is anonymous and pervasive, and can happen anytime and anywhere [37, 38]. Based on the intensity of threats and individual vulnerability of victims, cyberbullying may directly result in increased depression and suicidal behavior [35]. In the present study, the definition of cyberbullying was not determined; therefore, our findings were based on adolescents’ self-reported perceptions of cyberbullying. However, 1.8% of adolescents reported having experienced cyberbullying, of which 19.9% had attempted suicide and 52.0% had suicidal ideation (data not shown). A previous study investigated the association between level of Internet use and suicidal ideation or attempts in over 200,000 adolescents using a web-based survey; participants with a higher Internet addiction risk reported significantly higher suicidal ideation or suicide attempts [36]. Use of the Internet as a communication vehicle among children is increasing; therefore, guidance about cyberbullying should be provided in school health programs to help prevent possible suicidal behavior.

Another striking factor associated with suicidality in the present study was stress related to parental relationships. This factor showed a high OR as an associated factor among both junior high and high school students. However, the question concerning stress related to participants’ relationship with their parents did not define the meaning of “relationship with parents,” and it could therefore be widely interpreted. This stress might have included family support, family function, family conflict, communication, and other factors. Negative perceptions of family function or support have been reported as being significantly associated with suicidality in both community and clinical samples [6, 17, 19, 39–42]. Samm et al. [42] observed that self-reported satisfaction with family relationships and good communication with parents reduced the likelihood of suicidal thoughts in a non-clinical sample of adolescents. Susukida et al. [19] reported that individuals who perceived love from caregivers during childhood had a significantly lower prevalence of lifetime suicidal ideation than individuals without such perceptions; this finding was independent of whether the individuals lived with both biological parents during childhood. Susukida et al. [19] suggested that regardless of family structure, perceived support from caregivers during childhood is an important correlate of lifetime suicidal ideation. Similarly, in clinical samples, family discord and negative relationships with parents were associated with increased suicide risk among adolescents with depression [39–42]. Furthermore, suicidal ideation among children is associated with suicidal depression among their caregivers [31]. Evidence suggests that good relationships with parents lead to lower lifetime suicidal ideation. Therefore, it is important for health providers to assess individual family function, which may be a key factor to help prevent for suicidal ideation or attempted suicide among adolescents.

Another factor with a high OR in this study was stress related to sexual identity or intercourse—especially among high school students. Transgender adolescents have more suicide attempts than cisgender adolescents; gender identity subgroups show different rates of suicide attempts (female-to-male adolescents have the highest rate, at 50.8%) [43]. The prevalence of transgender individuals appears to be relatively low (1.4%) in Japan [44]; however, sexual minority adolescents remain at an increased risk for suicidality.

The number of adolescents who reported stress because of school records or their academic courses was high, and these stressors were significant risk factors for suicidality; however, the ORs were not higher than those for other factors. According to the Japanese government’s official announcement, poor school academic records and stress about academic courses were the most significant causes of adolescent suicide (9.9% of suicide cases were considered as having those conditions as a possible cause) [30]. In addition, that announcement indicated 8.8% of completed suicides were related to stressful relationships with parents, and the rate of overall bullying as a possible cause was only 1.8%; however, that investigation was based on assessment by each school principle or educational committee [30]. It is imperative that school health providers consider the discrepancy between the government’s official announcement and our results to develop and optimize preventive interventions.

The implication of these findings that showed many factors were associated with suicidality suggests that multiple factors simultaneously affect suicidality for adolescents rather than a single factor. Health providers and families need to keep this in mind to help prevent suicide among adolescents.

Another important finding in this study was that adolescents consulted different resources to manage stressors according to the type of stress. Our participants chose the Internet as a resource for worries or stress about the opposite sex, sexual identity, and relationships with parents. However, participants tended not to confide in their teachers, indicating reluctance to undertake help-seeking behavior. Our participants may have been concerned about confidentiality and stigma, which can prevent young people from seeking help. A growing body of evidence suggests that help-seeking behavior may influence the prevention of suicidal ideation or attempted suicide in adolescents [45–47]. Although friends, teachers, and family members are likely to be the greatest support resources for adolescents with suicidality, the majority of adolescents with suicidality do not seek help for their difficulties [48]. School-based mental health literacy educational programs are required to promote knowledge and help-seeking behavior among adolescents [49].

The present study had some limitations. The results must be considered in the context of potential response bias inherent in our survey process. First, ideally suicidal ideation and attempts would have been reviewed with two discrete questions. In our study, participants’ responses for suicidal ideation and suicide attempts were listed in one answer column to minimize the invasive effects on participants. In Japan, some educational committees still have the perception that talking about suicide and suicide prevention in the classroom as an educational program may increase the possibility of suicide in a vulnerable child. For this reason, we were obliged to minimize questions pertaining directly to suicidality. However, the questionnaire for suicidality was not validated. Second, we did not perform structured diagnostic and cognitive interviews with participants, and did not ask about depressive symptoms in the questionnaire. Therefore, we did not assess depressive disorders, which may be associated with the development of suicidality. Third, we did not evaluate participants’ socioeconomic status (e.g., income and education level) or family structure.

## Conclusion

In conclusion, we found the prevalence of suicidal ideation and attempted suicide in Japanese adolescents was 25.7 and 5.4%, respectively; these rates are similar to those observed in Western countries [3–7]. We simultaneously investigated various factors that were possibly associated with adolescents’ suicidality; we identified experience of cyberbullying as the most significant associated factor. The relationship between children and their parents is also important to consider in prevention efforts regarding suicidal behavior. Further research is needed about school- and home-based mental health education by health providers and families to facilitate help-seeking behavior among adolescents.

## Data Availability

The datasets used in this study are available from the corresponding author on reasonable request.
